# Current applications of intestinal organoids: a review

**DOI:** 10.1186/s13287-024-03768-3

**Published:** 2024-05-31

**Authors:** Tao Xiang, Jie Wang, Hui Li

**Affiliations:** 1 Department of Colorectal Surgery, The First Affiliated Hospital, Zhejiang University School of Medicine, Hangzhou, Zhejiang China; 2 State Key Laboratory for Diagnosis and Treatment of Infectious Diseases, National Clinical Research Center for Infectious Diseases, National Medical Center for Infectious Diseases, Collaborative Innovation Center for Diagnosis and Treatment of Infectious Diseases, The First Affiliated Hospital, Hangzhou, Zhejiang China; 3 Surgical Intensive Care Unit, The First Affiliated Hospital, Zhejiang University School of Medicine, Hangzhou, Zhejiang China

**Keywords:** Intestinal organoid, Intestinal transplantation, Inflammatory bowel disease, Drug-screening, Disease, Clinical trail

## Abstract

In the past decade, intestinal organoid technology has paved the way for reproducing tissue or organ morphogenesis during intestinal physiological processes in vitro and studying the pathogenesis of various intestinal diseases. Intestinal organoids are favored in drug screening due to their ability for high-throughput in vitro cultivation and their closer resemblance to patient genetic characteristics. Furthermore, as disease models, intestinal organoids find wide applications in screening diagnostic markers, identifying therapeutic targets, and exploring epigenetic mechanisms of diseases. Additionally, as a transplantable cellular system, organoids have played a significant role in the reconstruction of damaged epithelium in conditions such as ulcerative colitis and short bowel syndrome, as well as in intestinal material exchange and metabolic function restoration. The rise of interdisciplinary approaches, including organoid-on-chip technology, genome editing techniques, and microfluidics, has greatly accelerated the development of organoids. In this review, VOSviewer software is used to visualize hot co-cited journal and keywords trends of intestinal organoid firstly. Subsequently, we have summarized the current applications of intestinal organoid technology in disease modeling, drug screening, and regenerative medicine. This will deepen our understanding of intestinal organoids and further explore the physiological mechanisms of the intestine and drug development for intestinal diseases.

**Supplementary Information:**

The online version contains supplementary material available at 10.1186/s13287-024-03768-3.

## Introduction

The intestine, as a vital digestive organ, is considered to be the most complex organ in the human body [1]. In human physiological activities, it plays roles in the metabolism of nutrients, immune regulation, and mucosal barrier functions [2, 3]. Additionally, nowadays, with the advancement of research into the intestinal microbiota, numerous studies have discovered that the gut microbiota can exert several major physiological effects through the gut-liver axis, gut-lung axis, gut-brain axis, and gut-endocrine axis [4–6]. Intestinal epithelial cell damage, immune stress, and disorder of the gut microbiota can all contribute to the progression of intestinal diseases [7–9]. For example, Occludin downregulation limits intestinal epithelial cell apoptosis via Caspase-3 pathway in patients with Crohn's disease and ulcerative colitis [10]. In 2019, a study reported that stress can increase the expression of Gabra3 and induce colon inflammation in mice, while also impairing barrier function [11]. Another study analyzed metagenomic data from 3625 healthy women and found that the association between IBD and gut microbiota is age-specific [7]. Historically, research on intestinal diseases mainly relied on cell lines and animals, but both have significant drawbacks. Cell lines cannot well reflect the genetic characteristics of patients due to the lack of cell–cell and cell–matrix interactions in the intestinal microenvironment [12]. Animal studies, on the other hand, often face challenges such as high costs, long experimental cycles, and insurmountable species differences between humans and mice [13].

In 2009, the team led by Hans Clevers first used mouse Lgr5^+^ small intestinal stem cells to construct small intestinal organoids in vitro [14]. In 2013, organoids were named one of top ten technologies of the year by *Science*. Subsequently, after 5 years of rapid development, it was named the 2017 Method of the Year by *Nature*. The most significant advantage of organoids is that they are human-derived and nearly physiological, and can simulate multiple types of organ-specific disease states in vitro, such as tumors, primary sclerosing cholangitis and inflammatory bowel disease (IBD) [15–17]. Mechanistic research on disease pathology, efficacy of therapeutic intervention and potential off-target effects can effectively reduce the failure rate in the clinical development stage. It also plays a role in precision medicine and guides patients' clinical medication, which has huge potential commercial value [18, 19]. Using organoids as the keyword, the number of articles published on PubMed has grown rapidly, reaching 3400 in 2023. It can be said that the emergence of organoids represents a revolution in biology [20]. 

Intestinal organoids originate from a group of crypt base stem cells rich in Lgr5 [21]. These organoids possess a 3D cell structure, with the lumen side facing inward and the apical side facing the extracellular matrix [22]. The cell clusters within include all differentiated intestinal cell types, such as Paneth cells, absorptive enterocytes or colonocytes, goblet cells, and enteroendocrine cells [23]. Mouse intestinal organoids can grow in a medium containing the Wnt agonist R-spondin 1, epidermal growth factor (EGF), and Noggin [24]. For human-derived intestinal organoids, the culture medium needs to be supplemented with EGF, Noggin, R-spondin 1, Wnt3a, nicotinamide, ALK small molecule inhibitors, and MAPK inhibitors [25]. In addition to possessing various characteristics and cell types of intestinal epithelium, intestinal organoids also possess the ability for self-renewal, and physiological functions such as water and ion absorption and transport [26, 27]. They can also reflect the genetic characteristics of the individual from which they originated, offering advantages over cell line and animal experiments [28, 29]. There are significant differences in the genetics of organoids derived from different intestinal tissues. For example, GATA4 and Na^+^/H^+^ exchanger 3 activity are mainly expressed in the proximal small intestine, and apical sodium-dependent bile acid transporter (ASBT) and basolateral organic solute transporter beta subunit (OSTB) are mainly expressed in organoids in the distal ileum [30]. With the deepening of organoid research and the integration of multiple disciplines, researchers have successively developed patient-derived organoids (PDOs), tumor organoids, and engineered organoids (using microfluidics, chips, hydrogels, etc.) [31–34]. Currently, intestinal organoids are widely used in fields such as Crohn’s disease, colorectal cancer (CRC) disease modeling, drug screening, microbiota studies, and biomolecular delivery [35–37]. Additionally, in recent years, the development of regenerative medicine based on organoids as a new treatment for ulcerative colitis (UC) and short bowel syndrome (SBS) has become a focal point of interest [38–40].

**Fig. 1:**
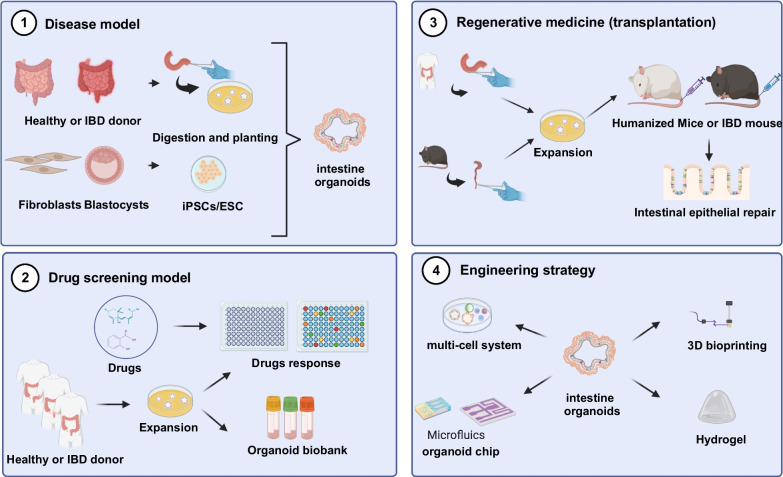
Applications of intestinal organoids. The various applications of intestinal organoids are as follows: (1) Developing in vitro models of intestinal organoids derived from adult tissues, pluripotent stem cells, or embryonic stem cells. (2) Constructing organoid from healthy individuals or patients for biobanks and drug screening. (3) Utilizing organoids for transplantation in the treatment of intestinal diseases. (4) Engineering modified organoids through interdisciplinary collaboration. *iPSC* induced pluripotent stem cell, *ESC* embryonic stem cell, *IBD* inflammatory bowel disease. Created with BioRender.com

This review aims to provide a fused perspective, offering a complete overview of the three main research directions of organoids and the attention-grabbing organoid engineering strategies (Fig. 1). Here, we not only clarified the current applications of organoids in disease models, drug screening, and organoid transplantation. But also, we are concerned about the bioengineering and materials fields, such as multi-cell models, organoid-on-chips, microfluics, 3D printing, hydrogel, etc. Organoid have arisen as prominent technologies for truly entering clinical practice and serving patients in the future.

## Research trend in intestinal organoid

### Data sources and methods

Firstly, we searched for research hotspots and progress on intestinal organoids on the Web of Science. Subsequently, bibliometric analysis and visualization were conducted using Vosviewer 1.6.20 software [41]. The criteria for including studies are as follows: (1) Research included in the core collection of Web of Science, including reviews and original articles. (2) The main research method or object is based on organoids, including various intestinal organoids from humans and mice/rat. (3) The period is from 2009 to January 2024.

According to the previously established retrieval strategy, 1343 articles were included in the study, including 1080 original articles, 263 review articles. The publication time has been concentrated in the past 7 years, and there has been a growing trend since 2009.

### Bibliometric analysis of publications

In the rapid progress of research on intestinal organoids, researchers in the Asia Pacific, European, and North American regions have made the most contributions. Among them, the United States (563 times, 41.921%) published the most documents, followed by China (204 times, 15.190%) and the Netherlands (193 times, 14.371%). Utrecht University (104 times, 7.744%) has published the most articles in this field, followed by Utrecht University Medical Center (95 times, 7.074%) and the Royal Netherlands Academy of Arts Sciences (77 times, 5.733%). As the founder of organoids, Clevers Hans has published the most literature (67 times, 4.989%). In addition, Sato T (33times, 2.457%), Spence JR (32times, 2.383%), Wells JM (23times, 1.713%), Beekman JM (17times, 1.266%) are the top five authors who published relevant literature.

**Fig. 2:**
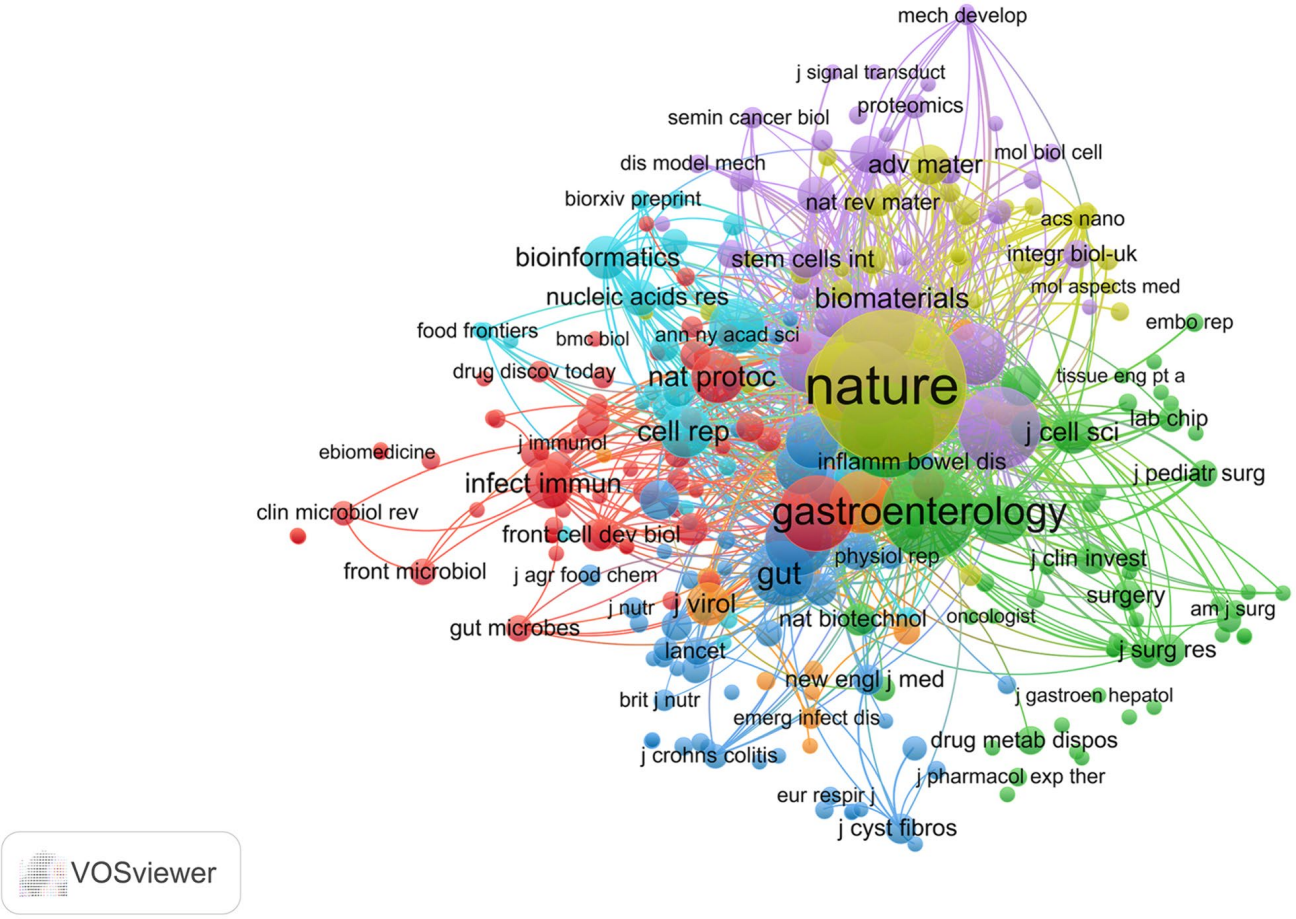
Co-citation analysis of intestinal organoids. The journals in the VOSviewer software are identified 674 journals, including 297 key journals. Created with VOSviewer software

Co-citation analysis can identify the most influential publication cluster in the field of intestinal organoids, which is crucial for understanding the latest knowledge dynamics in this field [42]. At the same time, this helps us determine the relevance of journals, assist researchers in selecting journals to submit and read, and identify areas for cross disciplinary research. Based on the results of co-citation analysis, we identified 674 journals, including 297 key journals (Fig. 2). As the forefront and hottest research topic, the study of intestinal organoids has been published multiple times in famous journals such as Nature (190 times), Cell (73 times), and Gastroenterology (72 times). In the field of intestinal organoids, these co-cited journals can be divided into 7 themes: Cell Biology (red), Gastroenterology Hepatology (green), Immunology and Microbiology (blue), Materials Science (yellow), Oncology (purple), Research Experimental Medicine and Food Science Technology (light blue) and Infectious Diseases (orange).

**Fig. 3:**
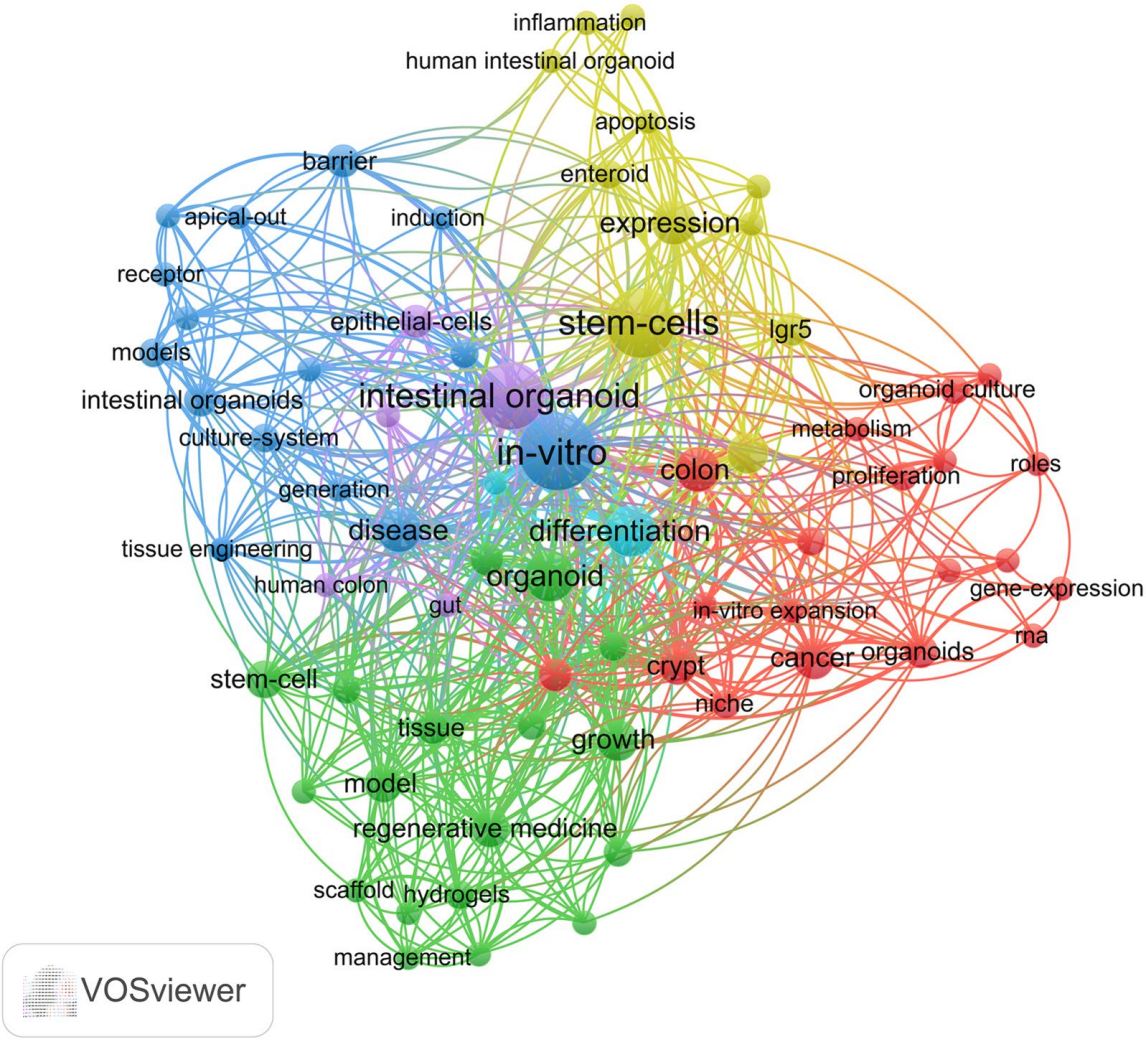
Co-word analysis of intestinal organoids. The keywords in the VOSviewer software are divided into 6 topic clusters. Created with VOSviewer software

Co-word analysis is a technique used to examine the actual content of publications themselves, as words that frequently appear simultaneously in research related to organoids have thematic relationships with each other [43]. In the co-word analysis, we obtained 371 keywords, including 70 main keywords (Fig. 3). The top 3 words are in vitro (21 times), stem cells (17 times), intestinal organoid (16 times). The keywords in the VOSviewer software are divided into 6 topic clusters. The most frequent topic cluster is organoid-based cell biology (red), which also includes exploration of molecular mechanisms and model construction of organoids in cancer. The second largest theme, engineered organoids (green), explores application of materials science in organoid culture and regenerative medicine. The third theme, organoid in vitro co-culture method (blue). The fourth theme, organoid-based molecular mechanism exploration and intestinal non-tumor disease simulation (yellow). The fifth theme is virus infection (purple) and the sixth theme is organoid-based differentiation (light blue).

Based on existing research results, it is reasonable to believe that the future trend of organoid research is organoid engineering and regenerative medicine applications (Fig. S1).

## Diseases model of intestinal organoid

In the realm of disease modeling, the most prevalent approach to date has been the use of cancer cell lines. However, commonly employed cell lines fail to encompass the cellular heterogeneity and genetic diversity observed within the intestinal milieu. Currently, widely utilized human intestinal epithelial cell lines such as Caco-2, HT-29, and SW-480, while cost-effective and readily available, do not offer the genetic instability or sensitivity to mutations inherent in native intestinal cells. Furthermore, these cell lines originate from tumor tissues or have undergone genetic editing, and they lack regional characteristics representative of the intestinal tract. Although animal models can better simulate the occurrence and development of diseases, they usually bring huge economic and time constraints as well as ethical issues, thus limiting their applicability in research. Clearly, we need a more appropriate disease model.

The advent of organoid models has heralded a transformative era in biomedical research. Traditional cell lines have proven inadequate in capturing the genetic disparities and functional variances specific to different segments of the digestive tract. Organoids derived from human tissues have effectively addressed this limitation. Kayisoglu, for instance, generated 42 human and murine organoid lines from adult and fetal gastric and intestinal segments, revealing highly specific expression profiles across various regions of the digestive tract [44]. Taking their investigation, a step further, Masi conducted a comprehensive analysis by comparing the whole transcriptomes of four preterm infant intestinal organoids and five adult intestinal organoids after separate co-cultivation with anaerobic bacteria. This in-depth research revealed that the genetic characteristics of preterm infant intestinal organoids differed significantly from those of adults. Furthermore, their responses to bacterial exposure exhibited marked distinctions, underscoring the unique genetic attributes and altered responsiveness of preterm infant gut organoids in the context of microbial interactions as compared to their adult counterparts [45].

Currently, organoid models have played a pivotal role in advancing research related to diseases such as IBD and CRC. In the pathogenesis of IBD, the intestinal epithelium serves as a mechanical barrier, a biochemical barrier, and plays a crucial role in antigen presentation. Kollmann, for instance, stimulated organoids and Caco-2 cells with a mixture of TNFα, IFN-γ, and IL-1ß [46]. The findings indicated that organoids outperformed the Caco-2 cell line, displaying a heightened sensitivity to inflammatory stimuli. They exhibited responses involving loss of cell–cell adhesion, redistribution of junctional proteins, compromised barrier function, and ultimately, epithelial cell death processes. Additionally, He leveraged the diverse cell types present in organoids and discovered that IL-22 does not directly control the regenerative capacity of crypt base stem cells but rather increases the number of Paneth cells [47]. Furthermore, Chiriac and colleagues, using organoids derived from IBD patients, identified that IL-20 could regulate the resolution of experimental colitis by modulating epithelial IFN/STAT2 signaling. They also targeted the uPA-uPAR interaction to enhance intestinal epithelial barrier integrity in IBD. These experiments underscore the capability of organoids derived from IBD patients to faithfully mimic the intestinal epithelium of IBD patients, thereby advancing research in the field of IBD [48, 49].

CRC is the fourth most lethal cancer in the world, killing nearly 900,000 people every year [50]. However, CRC still faces challenges in clinical treatment, most likely due to the lack of suitable models.

Numerous studies have demonstrated that CRC organoids retain the histopathological structure of their parent tumors, making them ideal models for studying cancer initiation, tumor invasion, and metastasis processes [51, 52]. Plattner, through the establishment of a sample library from CRC patients, conducted functional and spatial proteomic analyses, revealing intracellular and intercellular signal crosstalk in CRC [53]. Oncogenic mutations and microenvironmental signals regulate tumor cell fate. To comprehensively map how intracellular and extracellular signals jointly regulate cell fate, Qin conducted systematic single-cell analysis on 1107 colon organoids. It was found that oncogenic mutations dominate homeostatic differentiation by obstructing cell-extrinsic regulation of cell-fate plasticity [54]. Furthermore, human CRC organoids cultured in vitro have been shown to be transplantable in immunodeficient mice, typically engrafted in the mouse flank or kidney capsule, successfully recapitulating the tumor progression and metastasis processes [55, 56]. In 2020, a study demonstrated the use of intestinal organoids for forward genetic screening with a whole-genome CRISPR library to identify genes about TGF-β-mediated altered manifestations in CRC [57]. PDOs can facilitate personalized treatment strategies. For example, CRC patients exhibit high genetic heterogeneity, and there are significant differences in tumor microenvironmental cell populations, which can impact the effectiveness of therapeutics. In a recent study, Zapatero developed the “Trellis” approach to characterize CRC-PDOs and cancer-associated fibroblasts (CAFs), shedding light on mechanisms of CRC drug response or alterations in tumor microenvironmental cells [37]. In conclusion, the utilization of organoid-based disease modeling and analytical methods is gradually revolutionizing the research landscape in biomedicine, greatly accelerating progress in the study of diseases.

The rapid development of intestinal organoid provided a novel research platform for exploring the mechanisms of microbial–epithelial interactions and assessing probiotics and health foods. The most commonly used method for establishing co-culture models of organoids and microorganisms is microinjection [58, 59].

Using organoids, researchers have been able to study the complete processes of virus, bacteria, and eukaryotic parasites adhesion, invasion, infection, and replication within epithelial cells. For instance, in 2015, a study found that *Salmonella enterica serovar Typhimurium* could invade the epithelial barrier of intestinal organoids generated from human-induced pluripotent stem cells (hiPSCs) [60]. Nickerson [61] confirmed that *Shigella flexneri* effectively adhered to the cecum and colon in human intestinal organoid-derived epithelial monolayer model, with *Typhi* showing more efficient infection in the cecum than the ileum. Traditional cell models and animal models often cannot support the sustained and efficient replication of viruses, making organoids a significant advancement in this field. Intestinal organoids support viral infections, and post-differentiation, they express mature cell types found in the villous epithelium, including enterocytes, goblet cells, enteroendocrine cells, and Paneth cells, which is important for determining the specific tropism of viral enterocytes. For example, Finkbeiner and colleagues [62] used iPSC-derived human intestinal organoids to demonstrate that they support rotavirus replication. Ettayebi and colleagues [63] confirmed that human *norovirus* could infect intestinal organoids, selectively targeting enterocytes, which has important implications for preventing norovirus transmission and treating infections. Kolawole and colleagues [64] demonstrated that human intestinal organoids sourced from biopsies of different patients and intestinal regions could be infected by human astrovirus, with *VA1* infecting multiple cell types, including intestinal stem cells and mature enterocytes. Brevini and colleagues [65] used intestinal organoids to demonstrate that the Farnesoid X-Activated Receptor (FXR) inhibitor ursodeoxycholic acid can prevent SARS-CoV-2 infection by reducing Angiotensin Converting Enzyme 2 (ACE2) expression.

Furthermore, the rapid development of intestinal organoid chips has also opened new avenues for research related to microbiome-based therapies, probiotics, and health products [66]. The application of microfluidic technology enables precise control of oxygen gradients, growth factors, and fluid shear forces in the microenvironment of organoid chips, providing a more accurate simulation of luminal structure [32]. By adjusting the mechanical forces in the microenvironment of intestinal organoid chips, Grassart discovered that peristalsis influences the invasion of *Shigella flexneri* [67]. Jalili-Firoozinezhad used a microfluidic small intestine chip to control and real-time assess physiologically relevant oxygen gradients, offering unique insights into studying microbial differences under aerobic and anaerobic conditions [68]. In conclusion, the rapid development of intestinal organoid chips and their integration into microbial research has significantly advanced our understanding of microbial-epithelial interactions and holds great promise for the development of novel therapies, probiotics, and health products in the context of gastrointestinal health and diseases.

## Drug screening for intestinal organoids

Intestinal epithelial cells have the function of absorption and metabolism about drugs, nutrients, and water [69, 70]. Prior to the emergence of organoids, cell lines and animal models were the conventional platforms for drug screening, but both had significant limitations.

Primary intestinal cells can be isolated from human intestinal samples and retain the genetic characteristics and functions of their tissue of origin. They serve as excellent in vitro models for studying the molecular mechanisms of intestinal nutrition and gastrointestinal hormone secretion [71]. However, this system has drawbacks such as low reproducibility, short lifespan, and the inability to establish cell–cell contacts, which limits its applications.

Compared to primary cells, cell lines offer cost-effectiveness and time efficiency. Cell lines like Caco-2, for instance, are among the most commonly used platforms for drug screening. These cells feature well-differentiated brush borders on their apical surfaces, tight junctions, and express typical small intestinal microvillar hydrolases and nutrient transporters, making them popular in vitro models [72–74]. However, cell lines also have many disadvantages. Firstly, cell lines have tighter intercellular connections compared to human epithelial cells, exhibit distinct transporter proteins, lack complex epithelial cell functions and genetic characteristics, which can restrict the detection of certain drugs [75–77]. Secondly, many cell lines are derived from tumors or have undergone immortalization, making their gene expression profiling inconsistent with normal human intestinal epithelium [78]. Finally, cell lines cannot replicate the significant regional variations in the gastrointestinal tract. For instance, the pH in the small intestine ranges from 5 to 7, while in the large intestine, it ranges from 6 to 7.5. The small intestine primarily serves as the site for nutrient digestion and absorption, whereas the large intestine has a weaker nutrient transport capacity and primarily absorbs water, vitamins, and inorganic salts [79–81].

Rodents, dogs, and monkeys have also been used in drug metabolism studies. The metabolic processes, multi-organ responses, and potential adverse reactions of drugs in animals are important indicators for assessing drug efficacy and safety, and hold significant significance. However, as early as 1995, Kararli and colleagues found anatomical, physiological, and biochemical differences between humans and these experimental animals in the gastrointestinal tract [82]. For example, variations in the pH, composition, and content of digestive fluids can alter drug dissolution rates, solubility, and transport mechanisms. The types and quantities of gastrointestinal microbiota can also impact drug absorption via the oral route [56, 83, 84]. Additionally, the use of animals for drug screening and the assessment of efficacy and safety comes with high time and economic costs, as well as ethical concerns, all of which hinder the application of animal models.

Organoids, as predictive models for drug testing, offer unique advantages. If we conduct a PubMed search using the keywords “drug screening” and “organoid”, we can observe an explosive growth in research literature from 2012 to the present, with the majority of studies focused on drug screening in the context of CRC. Organoids can recapitulate the complexity of human tumors and predict drug toxicity responses in normal organs. Starting in 2015, researchers like Hans [52], Farin [85] and Luo [86] established PDOs models (41, 30, and 33 cases, respectively), which accurately represent tumor characteristics and can be used for high-throughput drug screening. Cartry et al. based on the establishment of 25 PDOs, screened 25 approved anticancer drugs. Their clinical application based on drug testing results showed a 75% sensitivity and specificity in predicting responses [87]. Mao, leveraging eight CRC organoids, developed a powerful organoid-based drug screening system. They tested 335 approved small molecule drugs and computationally predicted candidate drugs, successfully screening out 34 candidate drugs with efficacy against CRC organoids [88].

As research progressed, it was found that the 3D spherical structure of organoids limits the access of drugs and microorganisms to their lumens, thereby affecting assessment of intestinal permeability or drug absorption. Moreover, the presence of a relatively thick extracellular matrix gel around organoids may restrict the entry of drugs, nanoparticles, and microorganisms [89]. The intersection of microfluidic technology and organoid technology has given rise to intestinal chips, which have provided a robust and reliable system for drug screening. Intestinal chips technology can not only simulate the absorption, metabolism, and barrier functions of intestinal epithelial cells but also provide a more realistic intestinal microenvironment, including dynamic mechanical environments and oxygen gradients in the small intestine [90, 91]. For example, Kasendra developed an adult duodenal chip that, compared to common Caco-2 intestinal chips, can express CYP450 drug-metabolizing enzyme proteins and exhibits more similar gene expression to human duodenal tissue [92]. Wu [93] developed a high-throughput screening technology using a superhydrophobic microwell array chip (SMAR chip) for patient-derived tumor organoids, reducing reagent and sample consumption by over tenfold. Kulkarni and colleagues [94] introduced a method that combines organoids with intestinal chips. Organoids are implanted into the top compartment of the chip, while microvascular endothelial cells are integrated into the bottom compartment of the chip. This intestinal chip facilitates luminal exposure to Small molecule drugs, bacteria and other substances, promoting the research on intestinal microecology, intestinal barrier and transport [94].

Currently, a search on clinicaltrial.gov using the keyword “organoid” has revealed 13 clinical trials related to intestinal organoids after careful screening. As shown in Table 1, we classify the tables in chronological order and according to NCT numbers. Since 2017, clinical trials utilizing organoids as models have been conducted in the United States, China, France, and Germany. Among these trials, seven have explicitly stated their use for drug screening, while six are focused on disease modeling. This demonstrates the growing recognition of the potential of intestinal organoids in advancing research and clinical applications in the field of medicine. Companies specializing in microfluidic organs-on-chips/organoids-on-chips, such as Emulate Inc®, Mimetas®, and StemoniX®, have partnered with Johnson and Johnson, Roche, Takeda, Merck, and the FDA to validate the efficacy of their various products [95, 96]. Research and development costs limit innovation and contribute to the high prices of pharmaceuticals. Given that the development cost for a single drug is approximately one billion US dollars, the expenses associated with drug R&D and the risks of clinical trial failures present significant challenges for pharmaceutical companies [97]. It is under these circumstances that organ-on-a-chip technologies are endowed with the mission to innovate and redesign R&D processes, reduce costs, and potentially save lives [96]. Before intestinal organoids can be widely applied in the clinic, several key issues need to be emphasized: (1) Standardization and reproducibility of models. Variations exist in cell cytokine concentrations, activity, and Matrigel density during the culturing process; (2) Scaling up and automation of drug screening. Utilization of biotechnological approaches to construct cell chips and microfluidic technologies for ease of use; (3) Accuracy of disease models in organoid systems. Establishing multicellular organoid systems with more intestinal functionality remains challenging; (4) Ethical and regulatory concerns. While ethical issues surrounding organoids are relatively minor, organoids constructed from patient tissues still retain the genetic information of the patients, necessitating clear ethical guidelines. Collaborative efforts from clinicians, pharmacologists, bioengineering laboratories, and regulatory bodies are essential to translate laboratory achievements into clinical applications [98].

**Table 1:** Intestinal organoid models for drug testing in clinical trials

Organoid model	Objective	Status	Location	Study start	NCT number
Human intestinal organoid	The influence of nutrient antigens or therapeutic agents observed based on small intestinal human organoids	Recruiting	University of Erlangen-Nürnberg Medical School, Germany	2017-04-01	NCT03256266
Human intestinal organoid	These organoids will be used to study the biology of innervated sensory epithelial cells	Completed	Duke University, USA	2017-09-05	NCT02888587
Human colorectal cancer organoid	Drug screening of patient-derived organoids from advanced/recurrent/metastatic colorectal cancer culture	Recruiting	Chongqing University Cancer Hospital, China	2020-01-01	NCT05304741
Human gut organoid	Gut inflammation and gut-gut microbiome interactions in the pathogenesis of hypertension	Active, not recruiting	University of Florida, USA	2021-03-05	NCT04497727
Human colorectal cancer organoid	Tumor immune microenvironment involvement in colorectal cancer chemoresistance mechanisms	Not yet recruiting	University Hospital, Grenoble, France	2021-09-30	NCT05038358
Human colorectal organoid	Explore the consistency of drug sensitivity between primary colorectal cancer and liver metastases	Recruiting	Changhai Hospital, China	2022-01-01	NCT05183425
Human colorectal cancer organoid	Tumor tissue sampling for organoid development	Recruiting	University Hospital, Akershus, USA	2022-03-28	NCT05401318
Human intestinal organoid	Generate a biocollection of 3D intestinal models from digestive biopsies	Recruiting	Rennes University Hospital, France	2022-09-06	NCT05294107
Human intestinal organoid	Intestinal irradiation and inflammatory bowel disease from organoids biopsy	Recruiting	Institut National de la Santé Et de la Recherche Médicale, France	2022-09-19	NCT05425901
Human gastro-intestinal cancer organoid	Establishment of organoid cultures and in vitro sensitivity testing	Recruiting	Technische Universität Dresden, Germany	2022-12-08	NCT05652348
Human colorectal cancer organoid	Molecular Profiling & drug testing in tumor organoids	Not yet recruiting	Wuhan Union Hospital, China	2023-07-01	NCT05883683
Human colorectal cancer organoid	Chemotherapy based on organoid for colorectal cancer patient-derived tumor organoid drug sensitivity	Recruiting	Nanfang Hospital, Southern Medical University, China	2023-05-01	NCT05832398
Human colorectal cancer organoid	Using organoids for colorectal cancer drug sensitivity testing	Recruiting	China Medical University, China	2023-10-17	NCT06100016

In summary, human intestinal organoids exhibit characteristics that are closer to the complexity of the intestinal system compared to cell lines and primary cells. Compared with animal models, they have cost-effectiveness, time efficiency, and reduced ethical concerns.

## The status and challenges of intestinal organoids transplantation

Mounting evidence suggests that the intestinal epithelium plays a role in promoting host-microbe interactions, controlling mucosal immunity, coordinating nutrient cycling, and forming a mucosal barrier. Its repeated damage and repair are crucial in the pathogenesis of diseases such as primary sclerosing cholangitis (PSC), IBD, and celiac disease [99, 100]. For instance, bacterial translocation and T-cell immune responses can cause human colonic organoid epithelial damage, thereby promoting PSC disease progression [101, 102]. Besides, high expression of inflammatory factors such as IL-17A, IFN-γ, and TNF-a can promote apoptosis and necroptosis of intestinal epithelial cells [103, 104]. There is a long-term cycle of destruction and repair in the intestinal mucosal epithelium of IBD patients, which can cause chronic and recurrent inflammatory infiltration in the intestine and weaken the intestinal barrier function [105, 106]. Intestinal organoid transplantation to restore damaged intestinal epithelium offers a new treatment option for patients suffering from these diseases.

Organoid technology has enabled us to deepen our understanding of the function and structure of intestinal epithelial cells, thereby promoting the advancement of regenerative medicine. Since 2012, Shiro et al. have first depicted a serum-free expansion method for Lgr5^+^ colonic stem cells and repaired the intestinal epithelium of Rag2^−/−^ mice under dextran sulfate sodium (DSS) stimulation [107]. Currently, researchers are studying how to use natural intestinal submucosal tissue as a scaffold for direct organoid transplantation for diseases such as refractory UC. Nakanishi et al. [108] found that human induced pluripotent stem cell-derived intestinal organoids expressing intestinal-specific markers could be transplanted into the kidney capsule of mice, but not directly into the damaged intestines of mice. In the same year, to improve the intestinal epithelium in patients with UC, Satoshi and colleagues performed in situ transplantation of epithelial organoids into the colons of recipient mice through the anus, achieving repair of the intestinal epithelium in UC model mice [109]. Watanabe et al. [110] transplanted organoids derived from sulfur-rich mucin regions into the damaged recipient epithelial cells in mice with DSS-induced colitis. They discovered the transmissibility of sulfomucins in disease-related transplant models, contributing to the treatment of refractory UC [110]. SBS is a clinical syndrome resulting from intestinal resection due to reasons such as Crohn's disease, mesenteric ischemia, and surgical complications. Likewise, SBS lacks effective treatment methods. In 2021, Sato et al. found that transplanting ileal organoids into the colon of SBS rats can generate Small intestinalized colon (SIC) [111]. SIC can perform small intestine functions, reshape lymphatic vessel structures, and maintain muscular tissue regulated by nerve circuits and autonomous nervous control [38].

Another approach involves using decellularized scaffolds, animal/human matrices combined with intestinal organoids to create tissue engineered small intestine (TESI) [112]. In the past decade, in order to form more mature intestinal epithelia, good villi and crypts in TESI, researchers have conducted extensive studies. For example, they developed various biodegradable tissue scaffolds (PGA, PLLA, PCL) [113]; improved the sources of inter-scaffold matrices, ranging from rodent, hiPSC to human tissue-derived cells [114–116]; and enhanced TESI stem cell niches, intestinal villus height, and the proportion of proliferating epithelial cells in crypts through rhRSPO1 treatment, or overexpressing Fgf10 [117, 118]. Liu et al. compared TESI constructed from fetal mice, 5-day-old mice, 21-day-old mice, and 6-week-old adult rats as donors, and found that TESI derived from 5-day-old mice had the highest proliferative potential [119]. In 2021, Meran et al. constructed functional TESI using human jejunal organoids, jejunal fibroblasts, and HUVECS, a highly relevant step towards clinical translation [116]. However, current studies still face challenges in fully restoring neuromuscular functions and complete vascularization in TESI.

These studies all indicate the broad prospects of intestinal organoid for regenerative medicine. Here, we have summarized the intestinal organoid transplantation research available to date. As shown in Table 2, we classified the included studies according to the different sources of organoids used for transplantation and sorted them in chronological order. We excerpted the representative literature on intestinal organoid transplantation from 2006 to 2023. The hot research countries are the United States and Japan. Most researchers use the NSG IL2Rg-null mice model, and the main methods of intestinal organoid transplantation are to instill into the renal capsule through laparotomy or to instill into the colon and seal the anus. In addition, most of these studies aimed to study colonization of organoid. In summary, a considerable amount of research has been conducted on intestinal organoid derived from humans and mice, which has verified the effectiveness and safety of intestinal organoid transplants. In July 2022, a Japanese research team conducted the first autologous transplant using intestinal organoid cultured from healthy intestinal mucosal stem cells of a UC patient. This marks the true clinical application of intestinal organoid and represents another new milestone in the clinical use of intestinal organoid.

**Table 2:** Transplantation of intestinal organoids

Organoid type	Dosage and method	Disease	Receiver	Outcome	Location	Year	References
Rat/mouse neonatal small bowel organoids	5600 organoids; inject between the anterior and posterior leaves of the greater omentum	Malabsorption syndrome	Male Lewis rat or C57BL/6 mice	Orthotopic transplantation of intestinal mucosal organoids in rodents	United States	2006	[130]
Mouse crypt Organoids	500 organoids; instill into the colon and seal the anus	Ulcerative colitis	Immunocompromised Rag2^−/−^ mice + DSS	Transplanted organoids adhere to and cover superficially damaged tissue	Japan	2012	[107]
Human ESCs or iPSCs intestinal organoids	Not reported; through abdominal surgery to kidney capsule	Short bowel syndrome	NSG IL2Rg-null mice + intestinal resections	Colonization of intestinal organoids and display its good absorption, barrier and other functions	United States	2014	[131]
Human ESCs intestinal organoids	Not reported; through abdominal surgery to kidney capsule	NA	NSG IL2Rg-null mice	Transplanted organoids develop a structure more similar to the adult intestine	United States	2015	[134]
Human ESCs or iPSCs intestinal organoids	Not reported; through abdominal surgery to kidney capsule	NA	NSG IL2Rg-null mice	Synthetic hydrogel enables organoid survival, engraftment and wound repair	United States	2017	[135]
Human iPSCs intestinal organoids	Not reported; through abdominal surgery to kidney capsule	NA	NSG IL2Rg-null mice	Tracking transplanted organoid under the kidney capsule using fluorescence imaging	Korea	2018	[136]
Human intestinal organoids	200 organoids; through abdominal surgery to kidney capsule/lumen	NA/colitis model	NSG mice/NSG mice + DSS	Organoid colonization was successful	Korea	2020	[137]
Mouse colon organoids	1 × 106 dissociated organoids; through anus to colon by endoscopic procedure	Radiation proctitis	C57BL/6 mice	Organoid colonization was successful, reestablishing epithelial structure and integrity	Korea	2021	[138]
Human colon organoids	Dissociated organoids; through anus to colon	Ulcerative colitis	NSG IL2Rg-null mice	Transplantation of inflammation-depleted organoids into mouse colon mucosa	Japan	2021	[139]
Mouse rectal organoids	150–200 organoids; through anus to rectum	Radiation-induced rectal epithelial damage	C57BL/6 mice	Reduce rectal radiation damage	United States	2021	[140]
Human iPSC intestinal organoids	Not reported; through abdominal surgery to kidney capsule or reach the colon lumen via syringe	Ulcerative colitis	SCID-Beige mice/NSG mice + DSS	Intestinal organoids promote mucosal healing in mice model of acute colitis	Japan	2022	[108]
Human colon organoids	~ 1000 organoids; reach the colon lumen via syringe	Colitis model	C57BL/6 + DSS/RAG2^−/−^ + DSS	Organoid colonization was successful	Japan	2022	[109]
Human tumor organoid	50 dissociated organoids; injections of organoids into the submucosa of the colon by rigid endoscope	Colorectal cancer	Immunodeficient NSG + DSS	Orthotopically transplanted intestinal organoids form tumors in mice	Japan	2022	[141]
Human colon organoids	Dissociated organoids; through anus to colon	Ulcerative colitis	NSG IL2Rg-null mice	Therapeutic effect of telomerase activators on UC-model organoids	Japan	2022	[142]
Human iPSC intestinal organoids	Not reported; through abdominal surgery to bifurcating mesenteric vessels	NA	NSG IL2Rg-null mice	New culture method of human iPSC-induced intestinal organoids can colonize the mouse intestine	Japan	2022	[143]
Human ESC intestinal organoids	Not reported; through abdominal surgery to bifurcating mesenteric vessels	NA	NSG IL2Rg-null mice	Transplantation into the mouse mesentery is feasible and successful	United States	2023	[144]
Mouse intestinal organoids	5000 organoids per 200 μL; duodenal lumen by 1-mL syringe	Intestinal ischemia/reperfusion	C57BL/6 mice + intestinal I/R injury model	Organoid transplantation alleviates intestinal I/R injury via macrophages	China	2023	[145]
Human ESC intestinal organoids	Not reported; through abdominal surgery to kidney capsule	NA	NSG IL2Rg-null mice	Intestinal organoids maturation after transplantation resembles fetal intestinal development	United States	2023	[146]
Mouse cecum organoids	1000 organoids; inject into colon lumen	Ulcerative colitis	C57BL/6 mice + DSS	transplanted cecum organoids into the injured epithelium of distal colon	Japan	2023	[110]
Human ESC intestinal organoids	A single organoid; through abdominal surgery to kidney capsule	NA	NSG IL2Rg-null Tg (hIL3, hGM-CSF and hSCF) mice	Human immune cells combined with transplanted organoids	United States	2023	[147]
Rat IESCs organoids	350–400 organoids; tail vein injection	Cerebrovascular stroke	Older rats + middle cerebral artery occlusion	Organoid incorporation into the intestine restored stroke-induced intestinal malformations and reduced circulating levels of endotoxin LPS and IL-17A	United States	2023	[148]

*iPSC* induced pluripotent stem cell, *ESC* embryonic stem cell, *NA* not applicable, *DSS* dextran sulfate sodium, *IL-2R* interleukin-2 receptor, *LPS* lipopolysaccharide, *UC* ulcerative colitis, *Intestinal I/R* intestinal ischemia reperfusion, *RAG2* recombination activating protein 2

## Future prospects and conclusions

The discussed content highlights significant advancements of organoids in disease modeling, drug screening, and regenerative medicine, assessing the differences, strengths, and weaknesses of organoids and engineered organoids compared to primary cells, cell lines, and animals. Considering that the appropriate applications, advantages, and disadvantages of each model have been articulated in three separate sections, further elaboration here is omitted. We have categorized the models based on their differences and summarized their suitable applications, strengths, and weaknesses in Table 3. Furthermore, we will address some key challenges and potential directions for improvement in these fields.

**Table 3:** Characteristics of different intestinal models

Model	Representative type	Purpose	Advantages	Limitations	References
Immortalized intestinal cell lines	Caco-2; HT-29; SW-480 IEC-6; IEC-18; IEC-17	(1) Disease model	(1) Low cost (2) Less establishment time (3) No ethics issue	(1) Complexity (2) Genetically modified (3) Multicellular communication	[10, 149, 150]
Primary intestinal cells	Small intestinal epithelial cells; intestinal glial cells; colon lamina propria immune cells; colon epithelial cells	(1) Disease model (2) Regenerative medicine	(1) Low cost (2) Genetic characteristics (3) No ethics issue	(1) Complex procedure (2) Amplification and passage (3) Multicellular communication	[71, 151]
Intestinal organoids	Colon organoids; ileal organoids; rectal organoids	(1) Disease model (2) Drug research (3) Regenerative medicine	(1) 3D morphology (2) Genetic characteristics (3) No ethics issue	(1) Complex procedure (2) Moderate cost	[47, 109, 152]
Bioengineered intestinal organoids	Intestine‐chip; multi-organ chip; tissue engineered intestine	(1) Disease model (2) Drug research (3) Regenerative medicine	(1) Complexity (2) High throughput (3) No ethics issue	(1) Complex setup (2) Reproducibility (3) Moderate cost	[153, 154]
Animal*	DSS-fed mouse; TNBS-fed mouse; rats after partial colectomy; PDX model	(1) Disease model (2) Drug research	(1) Complexity (2) Multicellular communication	(1) High cost (2) Ethics issue (3) Low throughput	[155–157]

*DSS* dextran sulfate sodium, *TNBS* 2,4,6-trinitrobenzene sulfonic acid, *PDX* Patient-derived tumor xenograft

*Considering the current status of research, the animal model here refers to rodents

In the field of modeling intestinal diseases, iPSC-derived intestinal organoids have made it possible to simulate intestinal development and healthy homeostasis. PDOs accurately reflect the genetic heterogeneity and regional characteristics of diseases such as celiac disease [120], IBD [47], and CRC [121]. Additionally, intestinal organoids hold immense promise for studying the interactions between the intestinal tract and the microbiome (bacteria, viruses, etc.) [122]. In this field, it is important to note that the major limitation of organoids is their inability to replicate the intercellular and interorgan communication and exchange of substances. The integration of advanced technologies such as microfluidic systems, biomimetic scaffolds, and 3D printing is expected to facilitate the construction of biomimetic intestinal models [112, 123, 124]. Incorporating mesenchymal, vascular, neural, and epithelial tissues will bring these models closer to mimicking in vivo intestinal tissues. Furthermore, future challenges are anticipated to include finding a balance between standardization, repeatability, and reliability of organoids.

In the field of drug screening, the emergence of organoids has significantly alleviated the slow pace of drug development and resource wastage caused by the inability of traditional 2D cell and animal models to accurately predict human clinical trial outcomes. Multiple studies have confirmed the successful application of potential cancer chemotherapy drugs to clinical patients through organoid models, demonstrating the high predictability of organoids in simulating drug responses in the human system. The emergence of cutting-edge technologies such as 3D bioprinting or microfluidic chips raises the prospect of closely mimicking the tumor microenvironment [125]. However, it is important to note that current research often focuses on combinations of epithelial cells or fibroblasts. Vascularization, immunization, and multi-organ chips that link multiple organ modules (similar to the human body) appear to be better models for studying drug transport and metabolism [126, 127]. Additionally, the integration of AI technologies to construct relevant candidate drug libraries and analytical methods is expected to greatly enhance the efficiency of high-throughput drug screening [128, 129].

According to regenerative medicine, human organoids have the potential to be transplanted into immunodeficient mice with damaged intestines, becoming a potential source of transplantable tissues [130, 131]. Currently, there are two main methods for transplantation: direct transplantation of organoids and the construction of tissue-engineered small intestines, both of which can effectively repair damaged intestines [39, 111]. However, reconstructing the entire small intestine, including its complex lymphatic vascular system, remains challenging. Furthermore, while most studies report no tumor growth after the transplantation of tissue-derived organoids, ethical concerns related to human embryonic stem cell-derived organoids and the potential carcinogenicity of iPSCs must also be considered [132, 133].

To conclude, the development of organoids provides an unprecedented tool for human to study diseases. Intestinal organoids have already played a solid role in disease models, drug screening models, and regenerative medicine. By combining microfluidic technology, innovative biological support materials, and automated detection methods, we have more faithful that organoid technology will greatly accelerate the drug discovery for intestinal diseases and the innovation of treatment methods.

### Supplementary Information

Supplementary Material 1: Figure S1. Co-word analysis of intestinal organoids. The keywords in the VOSviewer software are divided into 6 topic clusters. Blue means close to 2009, yellow means close to 2023. Created with VOSviewer software.

## Data Availability

Not applicable.

## Abbreviations

ACE2: Angiotensin converting enzyme 2
ASBT: Apical sodium-dependent bile acid transporter
CAFs: Cancer-associated fibroblasts
CRC: Colorectal cancer
DSS: Dextran sulfate sodium
FXR: Farnesoid X-activated receptor
hiPSCs: Human-induced pluripotent stem cells
IBD: Inflammatory bowel disease
OSTB: Basolateral organic solute transporter beta subunit
PSC: Primary sclerosing cholangitis
PDOs: Patient-derived organoids
SMAR chip: Superhydrophobic microwell array chip
SBS: Short bowel syndrome
SIC: Small intestinalized colon
TESI: Tissue engineered small intestine
UC: Ulcerative colitis

## References

[1] Sharkey KA, Mawe GM (2023). The enteric nervous system. Physiol Rev.

[2] Farré R, Fiorani M, Abdu Rahiman S, Matteoli G (2020). Intestinal permeability, inflammation and the role of nutrients. Nutrients.

[3] Suzuki T (2020). Regulation of the intestinal barrier by nutrients: the role of tight junctions. Anim Sci J Nihon Chikusan Gakkaiho.

[4] Yan M, Man S, Sun B, Ma L, Guo L, Huang L, Gao W (2023). Gut liver brain axis in diseases: the implications for therapeutic interventions. Signal Transduct Target Ther.

[5] Perdijk O, Azzoni R, Marsland BJ (2023). The microbiome: an integral player in immune homeostasis and inflammation in the respiratory tract. Physiol Rev.

[6] Yang T, Richards EM, Pepine CJ, Raizada MK (2018). The gut microbiota and the brain-gut-kidney axis in hypertension and chronic kidney disease. Nat Rev Nephrol.

[7] Dong C, Guan Q, Xu W, Zhang X, Jin B, Yu S, Xu X, Xia Y (2023). Disentangling the age-related manner in the associations between gut microbiome and women's health: a multi-cohort microbiome study. Gut Microbes.

[8] Malik A, Sharma D, Aguirre-Gamboa R, McGrath S, Zabala S, Weber C, Jabri B (2023). Epithelial IFNγ signalling and compartmentalized antigen presentation orchestrate gut immunity. Nature.

[9] Manieri E, Tie G, Malagola E, Seruggia D, Madha S, Maglieri A, Huang K, Fujiwara Y, Zhang K, Orkin SH (2023). Role of PDGFRA+ cells and a CD55+ PDGFRALo fraction in the gastric mesenchymal niche. Nat Commun.

[10] Kuo WT, Shen L, Zuo L, Shashikanth N, Ong M, Wu L, Zha J, Edelblum KL, Wang Y, Wang Y (2019). Inflammation-induced occludin downregulation limits epithelial apoptosis by suppressing caspase-3 expression. Gastroenterology.

[11] Seifi M, Rodaway S, Rudolph U, Swinny JJG (2018). GABA receptor subtypes regulate stress-induced colon inflammation in mice. Gastroenterology.

[12] Shrestha J, Paudel KR, Nazari H, Dharwal V, Bazaz SR, Johansen MD, Dua K, Hansbro PM, Warkiani ME (2023). Advanced models for respiratory disease and drug studies. Med Res Rev.

[13] An Y, He Y, Ge N, Guo J, Yang F, Sun S (2023). Organoids to remodel SARS-CoV-2 research: updates, limitations and perspectives. Aging Dis.

[14] Sato T, Vries RG, Snippert HJ, van de Wetering M, Barker N, Stange DE, van Es JH, Abo A, Kujala P, Peters PJ (2009). Single Lgr5 stem cells build crypt-villus structures in vitro without a mesenchymal niche. Nature.

[15] Rodrigues J, Heinrich MA, Teixeira LM, Prakash J (2021). 3D in vitro model (r)evolution: unveiling tumor-stroma interactions. Trends Cancer.

[16] Kong L, Chen S, Huang S, Zheng A, Gao S, Ye J, Hua C (2024). Challenges and opportunities in inflammatory bowel disease: from current therapeutic strategies to organoid-based models. Inflamm Res Off J Eur Histamine Res Soc.

[17] Soroka CJ, Roberts SJ, Boyer JL, Assis DN (2021). Role of biliary organoids in cholestasis research and regenerative medicine. Semin Liver Dis.

[18] Hung SSC, Khan S, Lo CY, Hewitt AW, Wong RCB (2017). Drug discovery using induced pluripotent stem cell models of neurodegenerative and ocular diseases. Pharmacol Ther.

[19] Palasantzas V, Tamargo-Rubio I, Le K, Slager J, Wijmenga C, Jonkers IH, Kumar V, Fu J, Withoff S (2023). iPSC-derived organ-on-a-chip models for personalized human genetics and pharmacogenomics studies. Trends Genet TIG.

[20] Alhaque S, Themis M, Rashidi H (2018). Three-dimensional cell culture: from evolution to revolution. Philos Trans R Soc Lond B Biol Sci.

[21] Barker N, van Es JH, Kuipers J, Kujala P, van den Born M, Cozijnsen M, Haegebarth A, Korving J, Begthel H, Peters PJ (2007). Identification of stem cells in small intestine and colon by marker gene Lgr5. Nature.

[22] Almeqdadi M, Mana MD, Roper J, Yilmaz ÖH (2019). Gut organoids: mini-tissues in culture to study intestinal physiology and disease. Am J Physiol Cell Physiol.

[23] Middendorp S, Schneeberger K, Wiegerinck CL, Mokry M, Akkerman RD, van Wijngaarden S, Clevers H, Nieuwenhuis EE (2014). Adult stem cells in the small intestine are intrinsically programmed with their location-specific function. Stem Cells.

[24] Hirota A, AlMusawi S, Nateri AS, Ordóñez-Morán P, Imajo M (2021). Biomaterials for intestinal organoid technology and personalized disease modeling. Acta Biomater.

[25] Sato T, Stange DE, Ferrante M, Vries RG, Van Es JH, Van den Brink S, Van Houdt WJ, Pronk A, Van Gorp J, Siersema PD (2011). Long-term expansion of epithelial organoids from human colon, adenoma, adenocarcinoma, and Barrett's epithelium. Gastroenterology.

[26] Sato T, Ishikawa S, Asano J, Yamamoto H, Fujii M, Sato T, Yamamoto K, Kitagaki K, Akashi T, Okamoto R (2020). Regulated IFN signalling preserves the stemness of intestinal stem cells by restricting differentiation into secretory-cell lineages. Nat Cell Biol.

[27] Sugimoto S, Ohta Y, Fujii M, Matano M, Shimokawa M, Nanki K, Date S, Nishikori S, Nakazato Y, Nakamura T (2018). Reconstruction of the human colon epithelium in vivo. Cell Stem Cell.

[28] Mokry M, Middendorp S, Wiegerinck CL, Witte M, Teunissen H, Meddens CA, Cuppen E, Clevers H, Nieuwenhuis EE (2014). Many inflammatory bowel disease risk loci include regions that regulate gene expression in immune cells and the intestinal epithelium. Gastroenterology.

[29] Sato T, Clevers H (2013). Growing self-organizing mini-guts from a single intestinal stem cell: mechanism and applications. Science.

[30] Foulke-Abel J, In J, Yin J, Zachos NC, Kovbasnjuk O, Estes MK, de Jonge H, Donowitz M (2016). Human enteroids as a model of upper small intestinal ion transport physiology and pathophysiology. Gastroenterology.

[31] Vries RG, Huch M, Clevers H (2010). Stem cells and cancer of the stomach and intestine. Mol Oncol.

[32] Puschhof J, Pleguezuelos-Manzano C, Clevers H (2021). Organoids and organs-on-chips: insights into human gut–microbe interactions. Cell Host Microbe.

[33] Rajasekar S, Lin DSY, Abdul L, Liu A, Sotra A, Zhang F, Zhang B (2020). IFlowPlate-A customized 384-well plate for the culture of perfusable vascularized colon organoids. Adv Mater (Deerfield Beach, Fla).

[34] Howell KJ, Kraiczy J, Nayak KM, Gasparetto M, Ross A, Lee C, Mak TN, Koo BK, Kumar N, Lawley T (2018). DNA methylation and transcription patterns in intestinal epithelial cells from pediatric patients with inflammatory bowel diseases differentiate disease subtypes and associate with outcome. Gastroenterology.

[35] Tan H, Chen X, Wang C, Song J, Xu J, Zhang Y, Suo H (2023). Intestinal organoid technology and applications in probiotics. Crit Rev Food Sci Nutr.

[36] Hartwig O, Shetab Boushehri MA, Shalaby KS, Loretz B, Lamprecht A, Lehr CM (2021). Drug delivery to the inflamed intestinal mucosa—targeting technologies and human cell culture models for better therapies of IBD. Adv Drug Deliv Rev.

[37] Ramos Zapatero M, Tong A, Opzoomer JW, O'Sullivan R, Cardoso Rodriguez F, Sufi J, Vlckova P, Nattress C, Qin X, Claus J (2023). Trellis tree-based analysis reveals stromal regulation of patient-derived organoid drug responses. Cell.

[38] Endo R, Sugimoto S, Shirosaki K, Kato H, Wada M, Kanai T, Sato T (2023). Clinical challenges of short bowel syndrome and the path forward for organoid-based regenerative medicine. Regener Ther.

[39] Spurrier RG, Grikscheit TC (2013). Tissue engineering the small intestine. Clin Gastroenterol Hepatol Off Clin Pract J Am Gastroenterol Assoc.

[40] Okamoto R, Mizutani T, Shimizu H (2023). Development and application of regenerative medicine in inflammatory bowel disease. Digestion.

[41] van Eck NJ, Waltman L (2010). Software survey: VOSviewer, a computer program for bibliometric mapping. Scientometrics.

[42] Trujillo CM, Long TM (2018). Document co-citation analysis to enhance transdisciplinary research. Sci Adv.

[43] Zhang Y, Yin P, Liu Y, Hu Y, Hu Z, Miao Y (2022). Global trends and hotspots in research on organoids between 2011 and 2020: a bibliometric analysis. Ann Palliat Med.

[44] Kayisoglu O, Weiss F, Niklas C, Pierotti I, Pompaiah M, Wallaschek N, Germer CT, Wiegering A, Bartfeld S (2021). Location-specific cell identity rather than exposure to GI microbiota defines many innate immune signalling cascades in the gut epithelium. Gut.

[45] Masi AC, Fofanova TY, Lamb CA, Auchtung JM, Britton RA, Estes MK, Ramani S, Cockell SJ, Coxhead J, Embleton ND (2021). Distinct gene expression profiles between human preterm-derived and adult-derived intestinal organoids exposed to *Enterococcus*
*faecalis*: a pilot study. Gut.

[46] Kollmann C, Buerkert H, Meir M, Richter K, Kretzschmar K, Flemming S, Kelm M, Germer CT, Otto C, Burkard N (2023). Human organoids are superior to cell culture models for intestinal barrier research. Front Cell Dev Biol.

[47] He GW, Lin L, DeMartino J, Zheng X, Staliarova N, Dayton T, Begthel H, van de Wetering WJ, Bodewes E, van Zon J (2022). Optimized human intestinal organoid model reveals interleukin-22-dependency of paneth cell formation. Cell Stem Cell.

[48] Cheng Y, Hall TR, Xu X, Yung I, Souza D, Zheng J, Schiele F, Hoffmann M, Mbow ML, Garnett JP (2022). Targeting uPA-uPAR interaction to improve intestinal epithelial barrier integrity in inflammatory bowel disease. EBioMedicine.

[49] Chiriac MT, Hracsko Z, Günther C, Gonzalez-Acera M, Atreya R, Stolzer I, Wittner L, Dressel A, Schickedanz L, Gamez-Belmonte R (2023). IL-20 controls resolution of experimental colitis by regulating epithelial IFN/STAT2 signalling. Gut.

[50] Dekker E, Tanis P, Vleugels J, Kasi P, Wallace MJL (2019). Colorectal cancer. Lancet.

[51] Fujii M, Shimokawa M, Date S, Takano A, Matano M, Nanki K, Ohta Y, Toshimitsu K, Nakazato Y, Kawasaki K (2016). A colorectal tumor organoid library demonstrates progressive loss of niche factor requirements during tumorigenesis. Cell Stem Cell.

[52] van de Wetering M, Francies HE, Francis JM, Bounova G, Iorio F, Pronk A, van Houdt W, van Gorp J, Taylor-Weiner A, Kester L (2015). Prospective derivation of a living organoid biobank of colorectal cancer patients. Cell.

[53] Plattner C, Lamberti G, Blattmann P, Kirchmair A, Rieder D, Loncova Z, Sturm G, Scheidl S, Ijsselsteijn M, Fotakis G (2023). Functional and spatial proteomics profiling reveals intra- and intercellular signaling crosstalk in colorectal cancer. iScience.

[54] Qin X, Cardoso Rodriguez F, Sufi J, Vlckova P, Claus J, Tape CJ (2023). An oncogenic phenoscape of colonic stem cell polarization. Cell.

[55] Roper J, Tammela T, Cetinbas NM, Akkad A, Roghanian A, Rickelt S, Almeqdadi M, Wu K, Oberli MA, Sánchez-Rivera FJ (2017). In vivo genome editing and organoid transplantation models of colorectal cancer and metastasis. Nat Biotechnol.

[56] Kim N, Kwon J, Shin US, Jung J. Fisetin induces the upregulation of AKAP12 mRNA and anti-angiogenesis in a patient-derived organoid xenograft model. Biomed Pharmacother. 2023;167:115613 10.1016/j.biopha.2023.115613 37801904

[57] Ringel T, Frey N, Ringnalda F, Janjuha S, Cherkaoui S, Butz S, Srivatsa S, Pirkl M, Russo G, Villiger L (2020). Genome-scale CRISPR screening in human intestinal organoids identifies drivers of TGF-β resistance. Cell Stem Cell.

[58] Puschhof J, Pleguezuelos-Manzano C, Martinez-Silgado A, Akkerman N, Saftien A, Boot C, de Waal A, Beumer J, Dutta D, Heo I (2021). Intestinal organoid cocultures with microbes. Nat Protoc.

[59] Holokai L, Chakrabarti J, Broda T, Chang J, Hawkins JA, Sundaram N, Wroblewski LE, Peek RM, Wang J, Helmrath M (2019). Increased programmed death-ligand 1 is an early epithelial cell response to helicobacter pylori infection. PLoS Pathog.

[60] Forbester JL, Goulding D, Vallier L, Hannan N, Hale C, Pickard D, Mukhopadhyay S, Dougan G (2015). Interaction of *Salmonella*
*enterica* Serovar Typhimurium with intestinal organoids derived from human induced pluripotent stem cells. Infect Immun.

[61] Nickerson KP, Llanos-Chea A, Ingano L, Serena G, Miranda-Ribera A, Perlman M, Lima R, Sztein MB, Fasano A, Senger S (2021). A versatile human intestinal organoid-derived epithelial monolayer model for the study of enteric pathogens. Microbiol Spect.

[62] Finkbeiner SR, Zeng XL, Utama B, Atmar RL, Shroyer NF, Estes MK (2012). Stem cell-derived human intestinal organoids as an infection model for rotaviruses. MBio.

[63] Ettayebi K, Crawford SE, Murakami K, Broughman JR, Karandikar U, Tenge VR, Neill FH, Blutt SE, Zeng XL, Qu L (2016). Replication of human noroviruses in stem cell-derived human enteroids. Science.

[64] Kolawole AO, Mirabelli C, Hill DR, Svoboda SA, Janowski AB, Passalacqua KD, Rodriguez BN, Dame MK, Freiden P, Berger RP (2019). Astrovirus replication in human intestinal enteroids reveals multi-cellular tropism and an intricate host innate immune landscape. PLoS pathog.

[65] Brevini T, Maes M, Webb GJ, John BV, Fuchs CD, Buescher G, Wang L, Griffiths C, Brown ML, Scott WE (2023). FXR inhibition may protect from SARS-CoV-2 infection by reducing ACE2. Nature.

[66] Naumovska E, Aalderink G, Wong Valencia C, Kosim K, Nicolas A, Brown S, Vulto P, Erdmann KS, Kurek D (2020). Direct on-chip differentiation of intestinal tubules from induced pluripotent stem cells. Int J Mol Sci.

[67] Chen C, Jochems PGM, Salz L, Schneeberger K, Penning LC, van de Graaf SFJ, Beuers U, Clevers H, Geijsen N, Masereeuw R (2018). Bioengineered bile ducts recapitulate key cholangiocyte functions. Biofabrication.

[68] Jalili-Firoozinezhad S, Gazzaniga FS, Calamari EL, Camacho DM, Fadel CW, Bein A, Swenor B, Nestor B, Cronce MJ, Tovaglieri A (2019). A complex human gut microbiome cultured in an anaerobic intestine-on-a-chip. Nat Biomed Eng.

[69] Allaire JM, Crowley SM, Law HT, Chang SY, Ko HJ, Vallance BA (2018). The intestinal epithelium: central coordinator of mucosal immunity. Trends Immunol.

[70] Youhanna S, Lauschke VM (2021). The past, present and future of intestinal in vitro cell systems for drug absorption studies. J Pharm Sci.

[71] Dutton JS, Hinman SS, Kim R, Wang Y, Allbritton NL (2019). Primary cell-derived intestinal models: recapitulating physiology. Trends Biotechnol.

[72] Meunier V, Bourrié M, Berger Y, Fabre G (1995). The human intestinal epithelial cell line Caco-2; pharmacological and pharmacokinetic applications. Cell Biol Toxicol.

[73] Grosheva I, Zheng D, Levy M, Polansky O, Lichtenstein A, Golani O, Dori-Bachash M, Moresi C, Shapiro H, Del Mare-Roumani S (2020). High-Throughput screen identifies host and microbiota regulators of intestinal barrier function. Gastroenterology.

[74] Orth T, Neurath M, Schirmacher P, Galle PR, Mayet WJ (2000). A novel rat model of chronic fibrosing cholangitis induced by local administration of a hapten reagent into the dilated bile duct is associated with increased TNF-alpha production and autoantibodies. J Hepatol.

[75] Panse N, Gerk PM (2022). The Caco-2 model: modifications and enhancements to improve efficiency and predictive performance. Int J Pharm.

[76] Sambuy Y, De Angelis I, Ranaldi G, Scarino ML, Stammati A, Zucco F (2005). The Caco-2 cell line as a model of the intestinal barrier: influence of cell and culture-related factors on Caco-2 cell functional characteristics. Cell Biol Toxicol.

[77] Sun H, Chow EC, Liu S, Du Y, Pang KS (2008). The Caco-2 cell monolayer: usefulness and limitations. Expert Opin Drug Metab Toxicol.

[78] Fogh J, Fogh JM, Orfeo T (1977). One hundred and twenty-seven cultured human tumor cell lines producing tumors in nude mice. J Natl Cancer Inst.

[79] Cheng LK, O'Grady G, Du P, Egbuji JU, Windsor JA, Pullan AJ (2010). Gastrointestinal system. Wiley Interdiscip Rev Syst Biol Med.

[80] Jereb R, Opara J, Bajc A, Petek B (2021). Evaluating the impact of physiological properties of the gastrointestinal tract on drug in vivo performance using physiologically based biopharmaceutics modeling and virtual clinical trials. J Pharm Sci.

[81] Zietek T, Boomgaarden WAD, Rath E (2021). Drug screening, oral bioavailability and regulatory aspects: a need for human organoids. Pharmaceutics.

[82] Kararli TT (1995). Comparison of the gastrointestinal anatomy, physiology, and biochemistry of humans and commonly used laboratory animals. Biopharm Drug Dispos.

[83] Thomson AB, Hotke CA, Weinstein WM (1982). Comparison of kinetic constants of hexose uptake in four animal species and man. Comp Biochem Physiol A Comp Physio.

[84] Fagerholm U, Johansson M, Lennernäs H (1996). Comparison between permeability coefficients in rat and human jejunum. Pharm Res.

[85] Farin HF, Mosa MH, Ndreshkjana B, Grebbin BM, Ritter B, Menche C, Kennel KB, Ziegler PK, Szabó L, Bollrath J (2023). Colorectal cancer organoid-stroma biobank allows subtype-specific assessment of individualized therapy responses. Cancer Discov.

[86] Luo Z, Wang B, Luo F, Guo Y, Jiang N, Wei J, Wang X, Tseng Y, Chen J, Zhao B (2023). Establishment of a large-scale patient-derived high-risk colorectal adenoma organoid biobank for high-throughput and high-content drug screening. BMC Med.

[87] Cartry J, Bedja S, Boilève A, Mathieu JRR, Gontran E, Annereau M, Job B, Mouawia A, Mathias P, De Baère T (2023). Implementing patient derived organoids in functional precision medicine for patients with advanced colorectal cancer. J Exp Clin Cancer Res CR.

[88] Mao Y, Wang W, Yang J, Zhou X, Lu Y, Gao J, Wang X, Wen L, Fu W, Tang F (2023). Drug repurposing screening and mechanism analysis based on human colorectal cancer organoids. Protein Cell.

[89] Fatehullah A, Tan SH, Barker N (2016). Organoids as an in vitro model of human development and disease. Nat Cell Biol.

[90] Wang H, Xu C, Tan M, Su W (2023). Advanced gut-on-chips for assessing carotenoid absorption, metabolism, and transport. Crit Rev Food Sci Nutr.

[91] Cameron O, Neves JF, Gentleman E (2023). Listen to your gut: key concepts for bioengineering advanced models of the intestine. Adv Sci.

[92] Kasendra M, Luc R, Yin J, Manatakis DV, Kulkarni G, Lucchesi C, Sliz J, Apostolou A, Sunuwar L, Obrigewitch J (2020). Duodenum intestine-chip for preclinical drug assessment in a human relevant model. Elife.

[93] Wu Y, Li K, Li Y, Sun T, Liu C, Dong C, Zhao T, Tang D, Chen X, Chen X (2022). Grouped-seq for integrated phenotypic and transcriptomic screening of patient-derived tumor organoids. Nucleic Acids Res.

[94] Kulkarni G, Apostolou A, Ewart L, Lucchesi C, Kasendra M (2022). Combining human organoids and organ-on-a-chip technology to model intestinal region-specific functionality. J Vis Exp JoVE.

[95] Balijepalli A, Sivaramakrishan V (2017). Organs-on-chips: research and commercial perspectives. Drug Discov Today.

[96] Carvalho MR, Yan LP, Li B, Zhang CH, He YL, Reis RL, Oliveira JM (2023). Gastrointestinal organs and organoids-on-a-chip: advances and translation into the clinics. Biofabrication.

[97] Carvalho MR, Truckenmuller R, Reis RL, Oliveira JM (2020). Biomaterials and microfluidics for drug discovery and development. Adv Exp Med Biol.

[98] Ma C, Peng Y, Li H, Chen W (2021). Organ-on-a-chip: a new paradigm for drug development. Trends Pharmacol Sci.

[99] Camilleri M, Madsen K, Spiller R, Greenwood-Van Meerveld B, Van Meerveld BG, Verne GN (2012). Intestinal barrier function in health and gastrointestinal disease. Neurogastroenterol Motil Off J Eur Gastrointest Motil Soc.

[100] Parikh K, Antanaviciute A, Fawkner-Corbett D, Jagielowicz M, Aulicino A, Lagerholm C, Davis S, Kinchen J, Chen HH, Alham NK (2019). Colonic epithelial cell diversity in health and inflammatory bowel disease. Nature.

[101] Dean G, Hanauer S, Levitsky J (2020). The role of the intestine in the pathogenesis of primary sclerosing cholangitis: evidence and therapeutic implications. Hepatology (Baltimore, MD).

[102] Nakamoto N, Sasaki N, Aoki R, Miyamoto K, Suda W, Teratani T, Suzuki T, Koda Y, Chu PS, Taniki N (2019). Gut pathobionts underlie intestinal barrier dysfunction and liver T helper 17 cell immune response in primary sclerosing cholangitis. Nat Microbiol.

[103] Awane M, Andres PG, Li DJ, Reinecker HC (1999). NF-kappa B-inducing kinase is a common mediator of IL-17-, TNF-alpha-, and IL-1 beta-induced chemokine promoter activation in intestinal epithelial cells. J Immunol (Baltimore, Md: 1950).

[104] Appiah MG, Park EJ, Darkwah S, Kawamoto E, Akama Y, Gaowa A, Kalsan M, Ahmad S, Shimaoka M (2020). Intestinal epithelium-derived luminally released extracellular vesicles in sepsis exhibit the ability to suppress TNF-a and IL-17A expression in mucosal inflammation. Int J Mol Sci.

[105] Hausmann A, Steenholdt C, Nielsen OH, Jensen KB (2024). Immune cell-derived signals governing epithelial phenotypes in homeostasis and inflammation. Trends Mol Med.

[106] Emanuel E, Arifuzzaman M, Artis D (2024). Epithelial-neuronal-immune cell interactions: implications for immunity, inflammation, and tissue homeostasis at mucosal sites. J Allergy Clin Immunol.

[107] Yui S, Nakamura T, Sato T, Nemoto Y, Mizutani T, Zheng X, Ichinose S, Nagaishi T, Okamoto R, Tsuchiya K (2012). Functional engraftment of colon epithelium expanded in vitro from a single adult Lgr5^+^ stem cell. Nat Med.

[108] Nakanishi A, Toyama S, Onozato D, Watanabe C, Hashita T, Iwao T, Matsunaga T (2022). Effects of human induced pluripotent stem cell-derived intestinal organoids on colitis-model mice. Regener Ther.

[109] Watanabe S, Kobayashi S, Ogasawara N, Okamoto R, Nakamura T, Watanabe M, Jensen KB, Yui S (2022). Transplantation of intestinal organoids into a mouse model of colitis. Nat Protoc.

[110] Watanabe S, Ogasawara N, Kobayashi S, Kirino S, Inoue M, Hiraguri Y, Nagata S, Shimizu H, Ito G, Mizutani T (2023). Organoids transplantation as a new modality to design epithelial signature to create a membrane-protective sulfomucin-enriched segment. J Gastroenterol.

[111] Sugimoto S, Kobayashi E, Fujii M, Ohta Y, Arai K, Matano M, Ishikawa K, Miyamoto K, Toshimitsu K, Takahashi S (2021). An organoid-based organ-repurposing approach to treat short bowel syndrome. Nature.

[112] Levin DE, Barthel ER, Speer AL, Sala FG, Hou X, Torashima Y, Grikscheit TC (2013). Human tissue-engineered small intestine forms from postnatal progenitor cells. J Pediatr Surg.

[113] Liu Y, Nelson T, Chakroff J, Cromeens B, Johnson J, Lannutti J, Besner GE (2019). Comparison of polyglycolic acid, polycaprolactone, and collagen as scaffolds for the production of tissue engineered intestine. J Biomed Mater Res Part B Appl Biomater.

[114] Kitano K, Schwartz DM, Zhou H, Gilpin SE, Wojtkiewicz GR, Ren X, Sommer CA, Capilla AV, Mathisen DJ, Goldstein AM (2017). Bioengineering of functional human induced pluripotent stem cell-derived intestinal grafts. Nat Commun.

[115] Grant CN, Mojica SG, Sala FG, Hill JR, Levin DE, Speer AL, Barthel ER, Shimada H, Zachos NC, Grikscheit TC (2015). Human and mouse tissue-engineered small intestine both demonstrate digestive and absorptive function. Am J Physiol Gastrointest Liver Physiol.

[116] Meran L, Massie I, Campinoti S, Weston AE, Gaifulina R, Tullie L, Faull P, Orford M, Kucharska A, Baulies A (2020). Engineering transplantable jejunal mucosal grafts using patient-derived organoids from children with intestinal failure. Nat Med.

[117] Levin G, Zuber SM, Squillaro AI, Sogayar MC, Grikscheit TC, Carreira ACO (2020). R-Spondin 1 (RSPO1) increases mouse intestinal organoid unit size and survival in vitro and improves tissue-engineered small intestine formation in vivo. Front Bioeng Biotechnol.

[118] Torashima Y, Levin DE, Barthel ER, Speer AL, Sala FG, Hou X, Grikscheit TC (2016). Fgf10 overexpression enhances the formation of tissue-engineered small intestine. J Tissue Eng Regener Med.

[119] Liu Y, Wang Y, Chakroff J, Johnson J, Farrell A, Besner GE (2019). Production of tissue-engineered small intestine in rats with different ages of cell donors. Tissue Eng Part A.

[120] Porpora M, Conte M, Lania G, Bellomo C, Rapacciuolo L, Chirdo FG, Auricchio R, Troncone R, Auricchio S, Barone MV (2022). Inflammation is present, persistent and more sensitive to proinflammatory triggers in celiac disease enterocytes. Int J Mol Sci.

[121] Wang R, Li J, Zhou X, Mao Y, Wang W, Gao S, Wang W, Gao Y, Chen K, Yu S (2022). Single-cell genomic and transcriptomic landscapes of primary and metastatic colorectal cancer tumors. Genome Med.

[122] Allam-Ndoul B, Castonguay-Paradis S, Veilleux A (2020). Gut Microbiota and intestinal trans-epithelial permeability. Int J Mol Sci.

[123] Shukla P, Yeleswarapu S, Heinrich MA, Prakash J, Pati F (2022). Mimicking tumor microenvironment by 3D bioprinting: 3D cancer modeling. Biofabrication.

[124] Ramos P, Carvalho MR, Chen W, Yan LP, Zhang CH, He YL, Reis RL, Oliveira JM (2023). Microphysiological systems to study colorectal cancer: state-of-the-art. Biofabrication.

[125] Radhakrishnan J, Varadaraj S, Dash SK, Sharma A, Verma RS (2020). Organotypic cancer tissue models for drug screening: 3D constructs, bioprinting and microfluidic chips. Drug Discov Today.

[126] Shirure VS, Hughes CCW, George SC (2021). Engineering vascularized organoid-on-a-chip models. Annu Rev Biomed Eng.

[127] Schafer ST, Mansour AA, Schlachetzki JCM, Pena M, Ghassemzadeh S, Mitchell L, Mar A, Quang D, Stumpf S, Ortiz IS (2023). An in vivo neuroimmune organoid model to study human microglia phenotypes. Cell.

[128] Basile AO, Yahi A, Tatonetti NP (2019). Artificial intelligence for drug toxicity and safety. Trends Pharmacol Sci.

[129] Sarkar C, Das B, Rawat VS, Wahlang JB, Nongpiur A, Tiewsoh I, Lyngdoh NM, Das D, Bidarolli M, Sony HT (2023). Artificial intelligence and machine learning technology driven modern drug discovery and development. Int J Mol Sci.

[130] Avansino JR, Chen DC, Hoagland VD, Woolman JD, Stelzner M (2006). Orthotopic transplantation of intestinal mucosal organoids in rodents. Surgery.

[131] Watson CL, Mahe MM, Múnera J, Howell JC, Sundaram N, Poling HM, Schweitzer JI, Vallance JE, Mayhew CN, Sun Y (2014). An in vivo model of human small intestine using pluripotent stem cells. Nat Med.

[132] Rowe RG, Daley GQ (2019). Induced pluripotent stem cells in disease modelling and drug discovery. Nat Rev Genet.

[133] Crespo M, Vilar E, Tsai SY, Chang K, Amin S, Srinivasan T, Zhang T, Pipalia NH, Chen HJ, Witherspoon M (2017). Colonic organoids derived from human induced pluripotent stem cells for modeling colorectal cancer and drug testing. Nat Med.

[134] Finkbeiner SR, Hill DR, Altheim CH, Dedhia PH, Taylor MJ, Tsai YH, Chin AM, Mahe MM, Watson CL, Freeman JJ (2015). Transcriptome-wide analysis reveals hallmarks of human intestine development and maturation in vitro and in vivo. Stem Cell Rep.

[135] Cruz-Acuña R, Quirós M, Farkas AE, Dedhia PH, Huang S, Siuda D, García-Hernández V, Miller AJ, Spence JR, Nusrat A (2017). Synthetic hydrogels for human intestinal organoid generation and colonic wound repair. Nat Cell Biol.

[136] Jung KB, Lee H, Son YS, Lee JH, Cho HS, Lee MO, Oh JH, Lee J, Kim S, Jung CR (2018). In vitro and in vivo imaging and tracking of intestinal organoids from human induced pluripotent stem cells. FASEB J Off Publ Fed Am Soc Exp Biol.

[137] Lee H, Son MY (2020). Using bioengineered fluorescence for selective in vivo and ex vivo tracking of intestinal organoids derived from human pluripotent stem cells. Methods Mol Biol (Clifton, NJ).

[138] Jee J, Park JH, Im JH, Kim MS, Park E, Lim T, Choi WH, Kim JH, Kim WR, Ko JS (2021). Functional recovery by colon organoid transplantation in a mouse model of radiation proctitis. Biomaterials.

[139] Watanabe S, Nishimura R, Shirasaki T, Katsukura N, Hibiya S, Kirimura S, Negi M, Okamoto R, Matsumoto Y, Nakamura T (2021). Schlafen 11 is a novel target for mucosal regeneration in ulcerative colitis. J Crohn's Colitis.

[140] Tirado FR, Bhanja P, Castro-Nallar E, Olea XD, Salamanca C, Saha S (2021). Radiation-induced toxicity in rectal epithelial stem cell contributes to acute radiation injury in rectum. Stem Cell Res Ther.

[141] Welz L, Kakavand N, Hang X, Laue G, Ito G, Silva MG, Plattner C, Mishra N, Tengen F, Ogris C (2022). Epithelial X-box binding protein 1 coordinates tumor protein p53-driven DNA damage responses and suppression of intestinal carcinogenesis. Gastroenterology.

[142] Watanabe S, Hibiya S, Katsukura N, Kitagawa S, Sato A, Okamoto R, Watanabe M, Tsuchiya K (2022). Importance of telomere shortening in the pathogenesis of ulcerative colitis: a new treatment from the aspect of telomeres in intestinal epithelial cells. J Crohn's Colitis.

[143] Takahashi J, Mizutani T, Sugihara HY, Nagata S, Kato S, Hiraguri Y, Takeoka S, Tsuchiya M, Kuno R, Kakinuma S (2022). Suspension culture in a rotating bioreactor for efficient generation of human intestinal organoids. Cell RE Methods.

[144] Cortez AR, Poling HM, Brown NE, Singh A, Mahe MM, Helmrath MA (2018). Transplantation of human intestinal organoids into the mouse mesentery: a more physiologic and anatomic engraftment site. Surgery.

[145] Zhang FL, Hu Z, Wang YF, Zhang WJ, Zhou BW, Sun QS, Lin ZB, Liu KX (2023). Organoids transplantation attenuates intestinal ischemia/reperfusion injury in mice through L-Malic acid-mediated M2 macrophage polarization. Nat Commun.

[146] Singh A, Poling HM, Chaturvedi P, Thorner K, Sundaram N, Kechele DO, Childs CJ, McCauley HA, Fisher GW, Brown NE (2023). Transplanted human intestinal organoids: a resource for modeling human intestinal development. Development (Cambridge, England).

[147] Bouffi C, Wikenheiser-Brokamp KA, Chaturvedi P, Sundaram N, Goddard GR, Wunderlich M, Brown NE, Staab JF, Latanich R, Zachos NC (2023). In vivo development of immune tissue in human intestinal organoids transplanted into humanized mice. Nat Biotechnol.

[148] Mani KK, El-Hakim Y, Branyan TE, Samiya N, Pandey S, Grimaldo MT, Habbal A, Wertz A, Sohrabji F (2023). Intestinal epithelial stem cell transplants as a novel therapy for cerebrovascular stroke. Brain Behav Immun.

[149] Wang Y, Chang X, Zheng B, Chen Y, Xie J, Shan J, Hu X, Ding X, Hu X, Yu Q (2022). Protective effect of ganoderma atrum polysaccharide on acrolein-induced apoptosis and autophagic flux in IEC-6 Cells. Foods (Basel, Switzerland).

[150] Khan I, Mahfooz S, Saeed M, Ahmad I, Ansari IA (2021). Andrographolide inhibits proliferation of colon cancer SW-480 cells via downregulating notch signaling pathway. Anti-cancer Agents Med Chem.

[151] Rouch JD, Scott A, Lei NY, Solorzano-Vargas RS, Wang J, Hanson EM, Kobayashi M, Lewis M, Stelzner MG, Dunn JC (2016). Development of functional microfold (M) cells from intestinal stem cells in primary human enteroids. PLoS ONE.

[152] Qu M, Xiong L, Lyu Y, Zhang X, Shen J, Guan J, Chai P, Lin Z, Nie B, Li C (2021). Establishment of intestinal organoid cultures modeling injury-associated epithelial regeneration. Cell Res.

[153] Grant J, Lee E, Almeida M, Kim S, LoGrande N, Goyal G, Sesay AM, Breault DT, Prantil-Baun R, Ingber DE (2022). Establishment of physiologically relevant oxygen gradients in microfluidic organ chips. Lab Chip.

[154] Apostolou A, Panchakshari RA, Banerjee A, Manatakis DV, Paraskevopoulou MD, Luc R, Abu-Ali G, Dimitriou A, Lucchesi C, Kulkarni G (2021). A novel microphysiological colon platform to decipher mechanisms driving human intestinal permeability. Cell Mol Gastroenterol Hepatol.

[155] Mo J, Ni J, Zhang M, Xu Y, Li Y, Karim N, Chen W (2022). Mulberry anthocyanins ameliorate DSS-induced ulcerative colitis by improving intestinal barrier function and modulating gut microbiota. Antioxidants (Basel, Switzerland).

[156] Chen G, Ran X, Li B, Li Y, He D, Huang B, Fu S, Liu J, Wang W (2018). Sodium butyrate inhibits inflammation and maintains epithelium barrier integrity in a TNBS-induced inflammatory bowel disease mice model. EBioMedicine.

[157] Tian Y, Xu J, Li Y, Zhao R, Du S, Lv C, Wu W, Liu R, Sheng X, Song Y (2019). MicroRNA-31 reduces inflammatory signaling and promotes regeneration in colon epithelium, and delivery of mimics in microspheres reduces colitis in mice. Gastroenterology.

