# Supplementary material for: Current applications of intestinal organoids: a review
Source: Stem Cell Res Ther. 2024 May 31;15:155. doi: 10.1186/s13287-024-03768-3 (PMC11140936; doi:10.1186/s13287-024-03768-3)
Supplement: Supplementary file 1 — Supplementary Material 1: Figure S1. Co-word analysis of intestinal organoids. The keywords in the VOSviewer software are divided into 6 topic clusters. Blue means close to 2009, yellow means close to 2023. Created with VOSviewer software. [file 13287_2024_3768_MOESM1_ESM.docx]

**Current applications of intestinal organoids：A review**

**Tao Xiang**^a^**， Jie Wang**^b^**，Hui Li**^*c^

^a^**Department of Colorectal Surgery, The First Affiliated Hospital, Zhejiang University School of Medicine, Hangzhou, Zhejiang**

^b^**State Key Laboratory for Diagnosis and Treatment of Infectious Diseases, National Clinical Research Center for Infectious Diseases, National Medical Center for Infectious Diseases, Collaborative Innovation Center for Diagnosis and Treatment of Infectious Diseases, The First Affiliated Hospital, Zhejiang**

^c^**Surgical** [**Intensive**](file:///C:\Users\apple\AppData\Local\youdao\DictBeta\Application\7.1.0.0421\resultui\dict\?keyword=intensive) [**Care**](file:///C:\Users\apple\AppData\Local\youdao\DictBeta\Application\7.1.0.0421\resultui\dict\?keyword=care) [**Unit**](file:///C:\Users\apple\AppData\Local\youdao\DictBeta\Application\7.1.0.0421\resultui\dict\?keyword=unit)**, The First Affiliated Hospital, Zhejiang University School of Medicine, Hangzhou, Zhejiang**

***Corresponding author:**

**Hui Li**

**Surgical** [**Intensive**](file:///C:\Users\apple\AppData\Local\youdao\DictBeta\Application\7.1.0.0421\resultui\dict\?keyword=intensive) [**Care**](file:///C:\Users\apple\AppData\Local\youdao\DictBeta\Application\7.1.0.0421\resultui\dict\?keyword=care) [**Unit**](file:///C:\Users\apple\AppData\Local\youdao\DictBeta\Application\7.1.0.0421\resultui\dict\?keyword=unit)**, The First Affiliated Hospital, Zhejiang University School of Medicine, Hangzhou, Zhejiang**

E-mail: **sophie8564@zju.edu.cn**


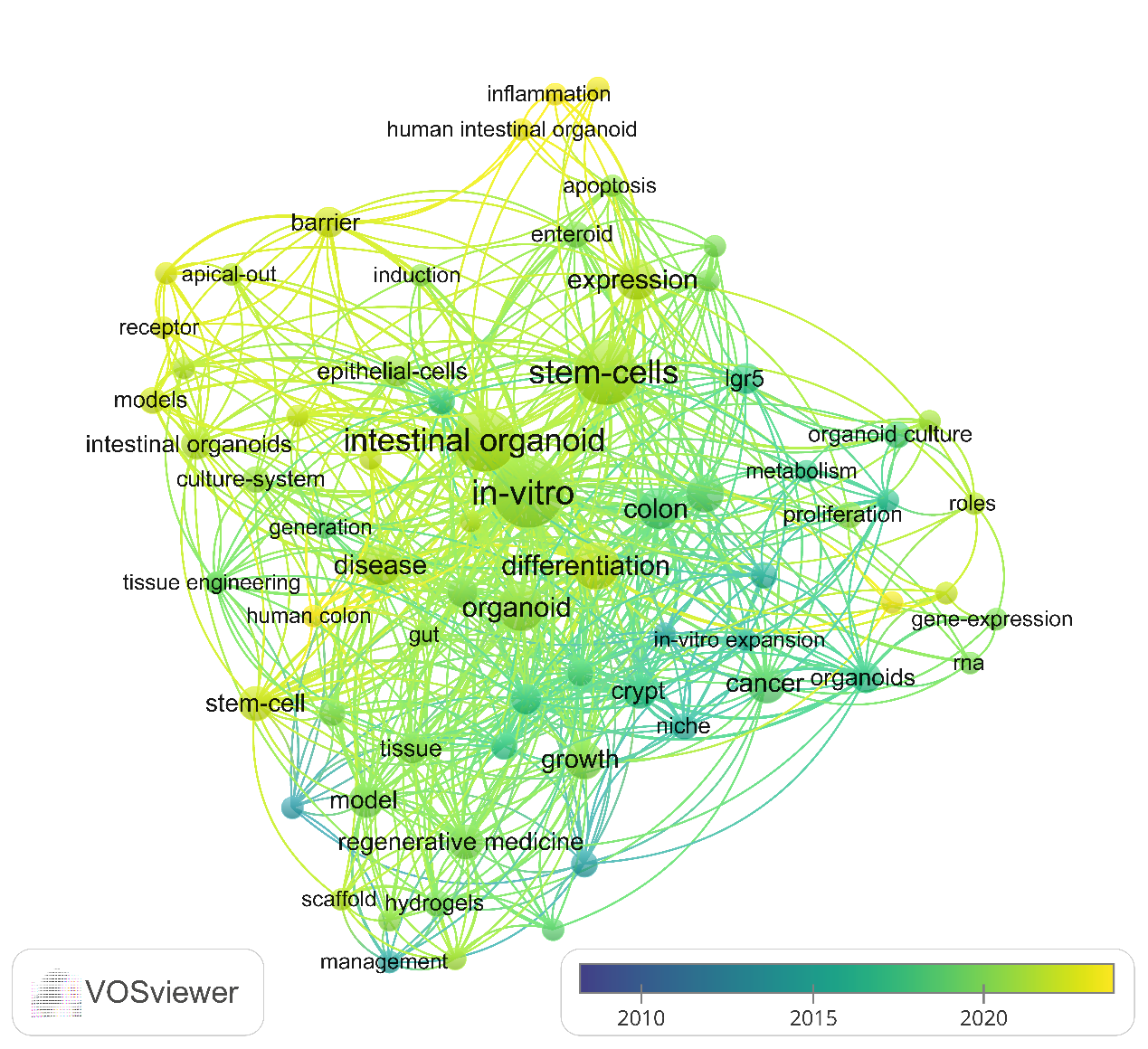


**Figure S1** Co-word analysis of intestinal organoids

The keywords in the VOSviewer software are divided into 6 topic clusters. Blue means close to 2009, yellow means close to 2023. Created with VOSviewer software.
